# Impairing flow-mediated endothelial remodeling reduces extravasation of tumor cells

**DOI:** 10.1038/s41598-021-92515-2

**Published:** 2021-06-23

**Authors:** Gautier Follain, Naël Osmani, Valentin Gensbittel, Nandini Asokan, Annabel Larnicol, Luc Mercier, Maria Jesus Garcia-Leon, Ignacio Busnelli, Angelique Pichot, Nicodème Paul, Raphaël Carapito, Seiamak Bahram, Olivier Lefebvre, Jacky G. Goetz

**Affiliations:** 1 Tumor Biomechanics, INSERM UMR_S1109, CRBS, 67000 Strasbourg, France; 2 Université de Strasbourg, 67000 Strasbourg, France; 3 Fédération de Médecine Translationnelle de Strasbourg (FMTS), 67000 Strasbourg, France; 4 Equipe Labellisée Ligue Contre le Cancer, Paris, France; 5 Present Address: Turku Bioscience Center,, University of Turku, Åbo Akademi University, 20520 Turku, Finland; 6 Present Address: UMR 5297, Interdisciplinary Institute for Neurosciences, CNRS Université de Bordeaux, 33076 Bordeaux, France

**Keywords:** Cellular imaging, Breast cancer, Cancer imaging, Target identification, Target validation, Cancer, Cell biology

## Abstract

Tumor progression and metastatic dissemination are driven by cell-intrinsic and biomechanical cues that favor the growth of life-threatening secondary tumors. We recently identified pro-metastatic vascular regions with blood flow profiles that are permissive for the arrest of circulating tumor cells. We have further established that such flow profiles also control endothelial remodeling, which favors extravasation of arrested CTCs. Yet, how shear forces control endothelial remodeling is unknown. In the present work, we aimed at dissecting the cellular and molecular mechanisms driving blood flow-dependent endothelial remodeling. Transcriptomic analysis of endothelial cells revealed that blood flow enhanced VEGFR signaling, among others. Using a combination of in vitro microfluidics and intravital imaging in zebrafish embryos, we now demonstrate that the early flow-driven endothelial response can be prevented upon specific inhibition of VEGFR tyrosine kinase and subsequent signaling. Inhibitory targeting of VEGFRs reduced endothelial remodeling and subsequent metastatic extravasation. These results confirm the importance of VEGFR-dependent endothelial remodeling as a driving force of CTC extravasation and metastatic dissemination. Furthermore, the present work suggests that therapies targeting endothelial remodeling might be a relevant clinical strategy in order to impede metastatic progression.

## Introduction

Metastatic colonization occurring during advanced tumorigenic progression is the main reason for cancer-related death^1^. It is currently well admitted that their development relies on biological and biophysical cues that influence their location and development^2^. Indeed, adhesion molecule repertoire^3,4^, vascular architecture^5–7^ and hemodynamics^2, 8,9^ impact the early steps of tumor cells dissemination. Several biochemical and physical parameters found in colonized organs such as growth factors, chemokines, stromal cell composition^10–13^ and matrix stiffness^14–16^ will permit the growth of secondary tumors.

In order to reach distant organs, tumor cells need to go through several rate-limiting steps including intravasation, blood-borne transport and extravasation^17^. Thus, a comprehensive understanding of these steps is necessary in order to design relevant pharmacological therapeutic strategies. Recently, using zebrafish embryo and mouse experimental metastasis models, we and others have demonstrated that blood flow-induced endothelial remodeling acts as a driving force to cell extravasation and subsequent micro-metastasis formation^9,18–20^. Specifically, we have shown that the endothelium wall actively remodels around arrested tumor cells in order to maintain vessel perfusion and thus actively promotes tumor cell extravasation. Such phenomenon relies on the ability of endothelial cells to protrude apically, migrate intravascularly and enwrap arrested CTCs, excluding them from the inner vasculature. Careful intravital imaging of mouse brain metastasis by triple negative breast cancer cells also revealed the presence of intraluminal endothelial plugs, which isolated invading cells from the circulation^9,20^. It was suggested that MMP enzymatic activity is required to resolve blood clot and maintain vessel perfusion through similar endothelial remodeling in mouse brain^21^ suggesting that tumor cells potentially hijack a physiological mechanism. However, the molecular mechanisms driving endothelial remodeling downstream of blood flow biomechanical cues remain elusive.

In the following report, we aimed at identifying the molecular programs that are transcriptionally activated downstream of flow forces, at values that are permissive to metastatic extravasation. Among others, activation of VEGFR signaling emerged as a potential major molecular signature of endothelial remodeling and thus an attractive target. Using two potent tyrosine-kinase and VEGFR inhibitor, one of them approved for clinical use to treat specific tumors such as renal cell carcinoma, gastro-intestinal stromal tumors or pancreatic neuroendocrine tumors^22^, we further demonstrate that VEGFR inhibition impairs extravasation of circulating tumor cells by blocking the endothelial remodeling. Thus, our findings confirm that endothelial remodeling is an essential step in metastatic extravasation and can be controlled at the molecular level using existing pharmacological tools^23^.

## Results

### Flow upregulates VEGF signaling pathway

**Figure 1:**
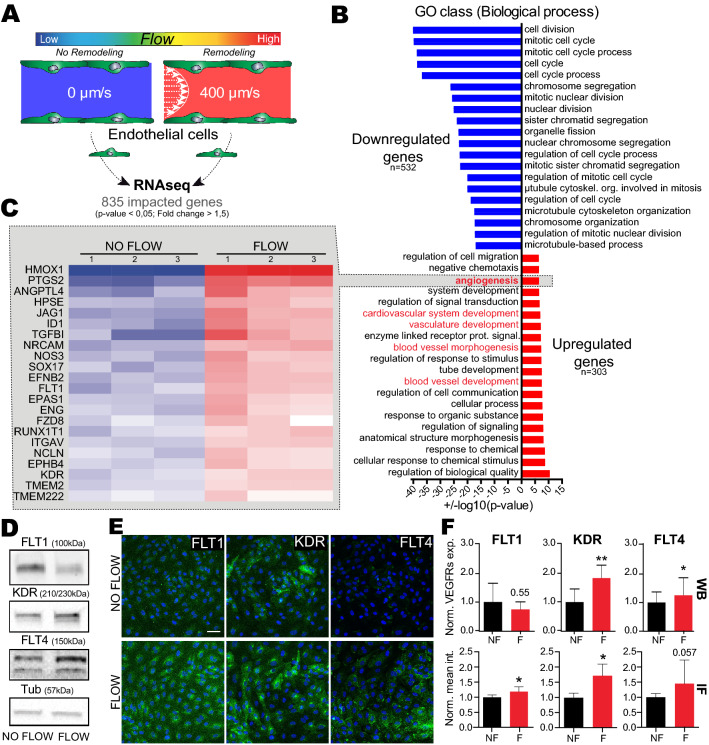
Flow favors expression of gene program driving vasculature remodeling, including VEGFRs. **(A) **Experimental setup: microfluidic experiment for RNA sequencing. (**B)** Results from global Gene Ontology (GO) analysis on ‘biological process’, showing most significantly impacted GO class. (**C)** Fold changes heatmap, based on GO class: Angiogenesis, showing significantly upregulated genes in flow condition compared to no flow. (**D)** Western blot quantification validating some of VEGFR RNAseq data. (**E)** Representative image of flow vs no flow immunofluorescence labelling of FLT1, KDR and FLT4 (green) with DAPI (blue). (**F)** Quantification associated with D and E, showing increased expression and signal intensity of VEGFRs. WB: N = 5, Mann Whitney test, IF: N = 4 with min 5 fields/exp, Mann Whitney test.

In order to identify signaling pathways that drive endothelial remodeling in dependence of permissive flow forces, we applied RNA sequencing on cultured primary endothelial cells (HUVEC) to identify transcriptional programs activated by previously identified flow profiles. We used previously described methods based on Human Umbilical Venous Endothelial Cells (HUVEC) culture in microfluidic channels^9^. The HUVEC monolayer was cultured for 16 h in static conditions or with medium perfusion at a flow rate of 400 µm/s (Fig. 1A). Such flow velocity was selected for two reasons: similar flow regime favors extravasation of tumor cells in zebrafish embryos, and it matches the flow rate measured in capillarylike vessels that we and others reported^9,24–27^. Total RNAs were then extracted, quantitative RNA sequencing was carried out and flow-responsive genes were clustered and functionally annotated using Gene Ontology (GO) databases.

Strikingly, endothelial cells switched their transcriptional programs from cell division and mitosis to cell migration/angiogenesis when exposed to flow (Fig. 1B and Fig S1A), as previously suggested^28^. Interestingly, a significant fraction of genes upregulated in response to flow is involved in vascular development, angiogenesis, and response to oxidative stress (Fig. 1B, Fig S1A and S4).** T**his is in accordance with previously described endothelial transcriptional response to shear stress^29^. Shear stress is indeed an essential biomechanical cue that controls the sprouting and migration of endothelial cells as well as vessel remodeling during angiogenesis^30–32^. Interestingly, intraluminal sprouting and migration of endothelial cells is initiated quickly upon CTC arrest in our models, the zebrafish embryo and the mouse brain^9^. We thus hypothesized that angiogenesis related genes might be elemental in driving endothelial remodeling during CTC extravasation. We thus focused on the GO class “Angiogenesis” (Fig. 1C) and applied a stringent threshold to our list of significantly impacted gene (with fold changes > 1.5 and p-value < 0.05). Among others, we identified FLT1 (VEGFR1) and KDR (VEGFR2), two VEGF receptors (VEGFR) that were strongly upregulated in endothelial cells subjected to our reference flow velocity (400 µm/s). Using RT-qPCR, we confirmed a specific two-fold increase in gene expression of FLT1 and KDR upon flow stimulation while non-VEGFR endothelial genes (Notch and LAMA4) where unaffected (Fig S1B,C). Using similar conditions, we assessed the protein levels of all three VEGFRs (FLT1, KDR and FLT4 (VEGFR3)) using western-blot and immunofluorescence (Fig. 1D–F, S5). Expression levels of KDR are significantly and consistently increased in response to flow. Expression levels of FLT1 and FLT4 also increase in response to flow, yet they display a heterogeneous response (Fig. 1D–F, S5). We sought out to confirm these results in vivo using the zebrafish embryo endothelium as a model. *Tg(fli1a:EGFP)* zebrafish embryos at 48 h post-fertilization (hpf) expressing EGFP in the endothelium were treated for 14 h with lidocaine or vehicle to decrease the heart pacemaker activity and thus blood flow as we previously described ^9^. Embryos were dissociated and GFP + endothelial cells were FACS sorted. Total RNAs of the endothelium were extracted and the expression levels of KDR and KDRL, the 2 zebrafish orthologs of VEGFR2, in vehicle and lidocaine-treated embryos was assessed by RT-qPCR. We observed that decreasing blood flow led to a decrease in both KDR and KRDL expression (Fig S1D). Altogether, these data suggest that permissive flow profiles for endothelial remodeling and subsequent extravasation of CTCs drive expression of VEGFRs, both at the RNA and protein levels. This prompted us to test the role of VEGFR pathways on the flow-dependent endothelial remodeling leading to extravasation of tumor cells.

### Inhibition of the VEGF pathway in vitro suppresses endothelial remodeling

**Figure 2:**
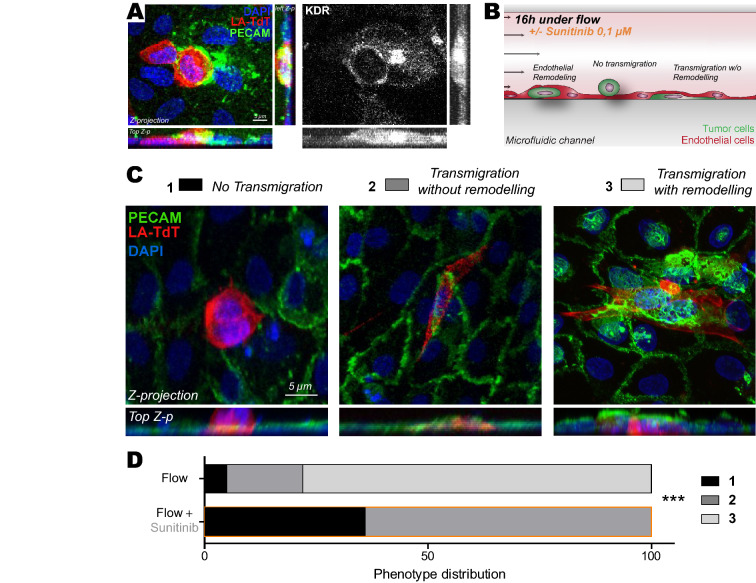
Inhibition of VEGFRs with sunitinib impairs endothelial remodeling in vitro. **(A)** Immuno-labelling pictures showing the presence of KDR (white) at the site of endothelial remodeling (Assessed with PECAM enrichment (green) around tumor cell (red)). Z-projections and two z-projection from side and top view are shown. (**B)** Experimental setup: microfluidic channel for endothelial remodeling assay**. (C)** Representative images of all steps: 1-No transmigration, 2-Transmigration without remodeling evidence, 3-Transmigration with remodeling. Quantification of the normalized number of transmigrating cells and remodeling activity. (**D)** Phenotypic distribution of CTCs attached to endothelial layers exposed to flow and treated with vehicle or sunitinib. N: flow/vehicle = 88, flow/sunitinib = 150, Fischer test.

Based on our previous results, we hypothesized that VEGFR signaling could trigger flow-dependent endothelial remodeling, and subsequent metastasis, and decided to pharmacologically impair it. Here, we decided to employ pharmacological inhibition using a clinically approved drug, sunitinib (Sutent or SU11248), for its known anti-angiogenic properties through VEGFRs, among others, as we further confirmed that several of its known targets were upregulated in response to flow in HUVEC cells (Fig S1C). Our goal, at this stage, was to demonstrate that molecular inhibition of VEGFR, using treatment regimes that do not cause major vascular remodeling effects but rather target flow-mediated intraluminal endothelial remodeling^9^, is a possible avenue for impairing metastatic extravasation. We first relied on our previously published microfluidic-based experimental model^9^. This system allows to recapitulate major features of the metastatic cascade such as arrest, adhesion and extravasation^33^. In brief, D2A1 cancer cells (mouse mammary carcinoma) are loaded into a microfluidic channel containing a monolayer of endothelial (HUVEC) cells and left to adhere for 10 min. Using a peristaltic pump, cells were then submitted to flow with a speed of 400 µm/s (i.e. permissive for CTC adhesion and activating endothelial response)^9^. With this approach, we confirmed that VEGFR2, a VEGFR isoform we found consistently overexpressed at the RNA and protein level, is recruited at the plasma membrane of endothelial cells undergoing remodeling during CTC extravasation in vitro (Fig. 2A). Using similar microfluidics where endothelial cells were subjected to VEGFR inhibition using sunitinib prior to CTC perfusion, we next assessed whether such treatment would impact endothelial remodeling and CTC transmigration (Fig. 2B). The microfluidic channels were perfused with tumor cells (CTCs) that adhered to endothelial cells and further flow-stimulated for 16 h in the presence of vehicle or sunitinib. As previously described^9^, flow stimulation favored transmigration through the endothelial cells monolayer of ~ 95% of the cancer cells. Among these, the vast majority (~ 80%) of the cells that had crossed the endothelial wall did so through endothelial remodeling (Fig. 2C,D). VEGFR inhibition through mild sunitinib treatment significantly decreased the number of tumor cells that could cross the endothelial barrier and suppressed the endothelial remodeling activity (Fig. 2C,D). In conclusion, flow stimulates VEGFR-dependent endothelial remodeling and subsequent metastatic extravasation in microfluidic artificial environments in vitro.

### Inhibition of the VEGFR pathway blocks early endothelial remodeling in zebrafish embryos

We next wondered whether such mechanism would occur in realistic hemodynamic situations. Zebrafish is an easy-to-use experimental metastasis model which is fully compatible with intravital imaging and chemical compound screening which can be directly added into the water^34,35^. In addition, we and others have shown that tumor cells engage in intraluminal vascular remodeling during metastatic extravasation^9,18,19^. We thus set out to test the involvement of VEGFR signaling, and its potential inhibition, in endothelial remodeling in vivo, using intravital imaging of intravascularly arrested tumor cells in zebrafish embryos.

**Figure 3:**
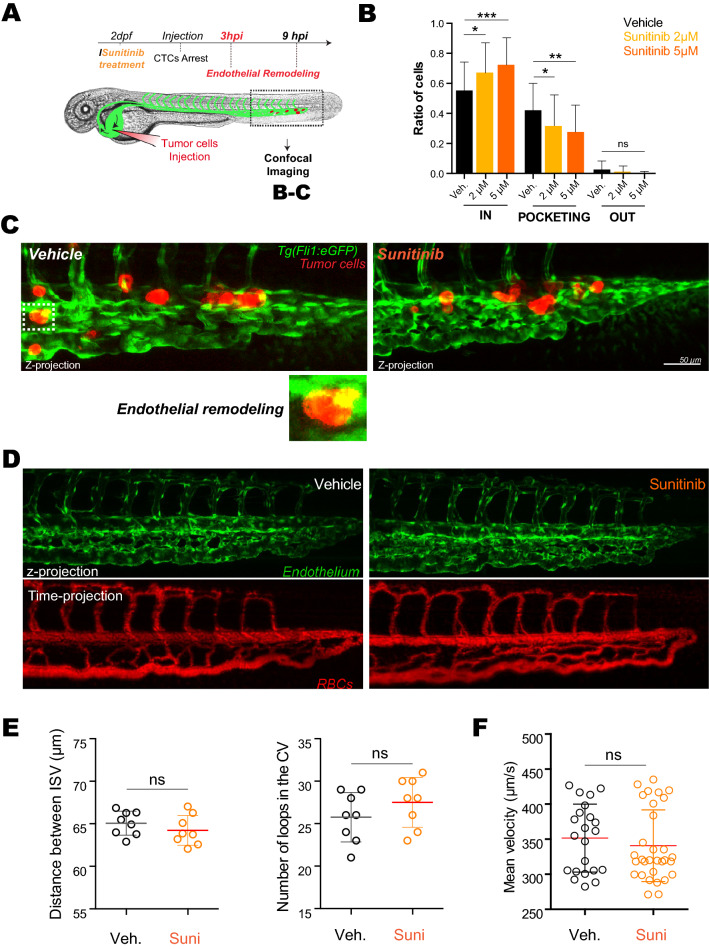
Inhibition of VEGFRs with sunitinib reduces endothelial remodeling in zebrafish embryos. **(A)** Experimental setup used: zebrafish are imaged at 3 hpi (hours post-injection). (**B)** Quantification of intravascular, remodeling and extravasated cells 3hpi. N cells: vehicle = 622, sunitinib 2 µM = 375, sunitinib 5 µM = 254. N embryos : vehicle = 62, sunitinib 2 µM = 34, sunitinib 5 µM = 27, Kruskal–Wallis test followed by Dunn's multiple Comparison test. **(C)** Representative image of arrested D2A1 cells in the caudal plexus of vehicle or sunitinib treated embryos. Vehicle: DMSO. (**D)** Representative confocal image of the caudal plexus architecture (upper panel) and blood flow (lower panel, RBC = red blood cells) 3 h post-treatment with sunitinib vs vehicle in *Tg(fli1a:EGFP;gata1:DsRed)*. (**E)** Quantification of the effect of sunitinib on vessel architecture after 3 h of treatment with vehicle or 5 µM of sunitinib, N = 8, Student t test. (**F**) Blood flow perfusion profiles of the same embryos (from **D**) and quantification of the blood flow velocity in the caudal plexus of embryos in both conditions after 3 h of treatment with vehicle or 5 µM of sunitinib. N embryos: vehicle = 8, sunitinib 5 µM = 10. Mann Whitney test.

Using our intravital imaging-based experimental metastasis model in zebrafish, we next injected tumor cells directly in the circulation through the duct of Cuvier of 48hpf embryos^36^ and quantitatively addressed the effect of sunitinib on extravasation in vivo (Fig. 3A). We classified the behavior of injected tumor cells into three populations at different stages of this process, whose dynamics has been characterized in our previous work : “intravascular”, “pocketing” (i.e. in the process of extravasation through endothelial remodeling) and “extravasated”^9^. After only 3 h post-injection (hpi), tumor cells are stably adhered to the endothelium, which starts to remodel in order to restore blood flow and favor metastatic extravasation (Fig. 3B,C). Quantification performed over a large number of embryos shows that short sunitinib treatment significantly impairs endothelial remodeling in a dose-dependent manner. Indeed, VEGFR inhibition increases the ratio of intravascular cells and decreases the number of pocketing events (Fig. 3B,C).

Interestingly, we carefully documented that such treatment regime, i.e. short and potent, has no effect on the overall vasculature and hemodynamics properties.

To rule out side-effects, we first controlled whether a short (3 h) sunitinib treatment (5 µM) would impair the vascular system and hemodynamics of *Tg(fli1a:EGFP; gata1:DsRed)* zebrafish embryos at 48 h post-fertilization (hpf) expressing EGFP in the endothelium and RFP in red blood cells (Fig. 3D). Pharmacological treatments of the embryos had mostly no impact on the vascular architecture of embryos, with only the most caudal inter-somitic vessels (ISVs) failing to lumenize in a few embryos (Fig. 3D). Anatomical analysis and quantification of the vascular system revealed that the vascular architecture was mostly unperturbed upon sunitinib treatment (Fig. 3E). Furthermore, using automated tracking analysis of the red blood cells (DsRed-positive) that allows to extract and quantify the mean velocity of the blood flow, we could not detect any major effect of VEGFR inhibition of overall hemodynamics (Fig. 3F). Altogether, this set of control experiments demonstrates that short sunitinib treatment does not impact the pre-established vascular system, allowing us to investigate whether it would perturb endothelial remodeling and subsequent metastasis.

Taken together, these in vivo experiments demonstrate that short inhibition of VEGFR, at regimes that target intraluminal remodeling and do not cause major vascular defects, is capable of impairing local endothelial remodeling upon CTC arrest without major deleterious effects on hemodynamics and vascular architecture. Thus, VEGFR signaling pathway is involved in the early steps of endothelial remodeling.

### Inhibition of the VEGF pathway impairs extravasation of metastatic cells in zebrafish embryos

**Figure 4:**
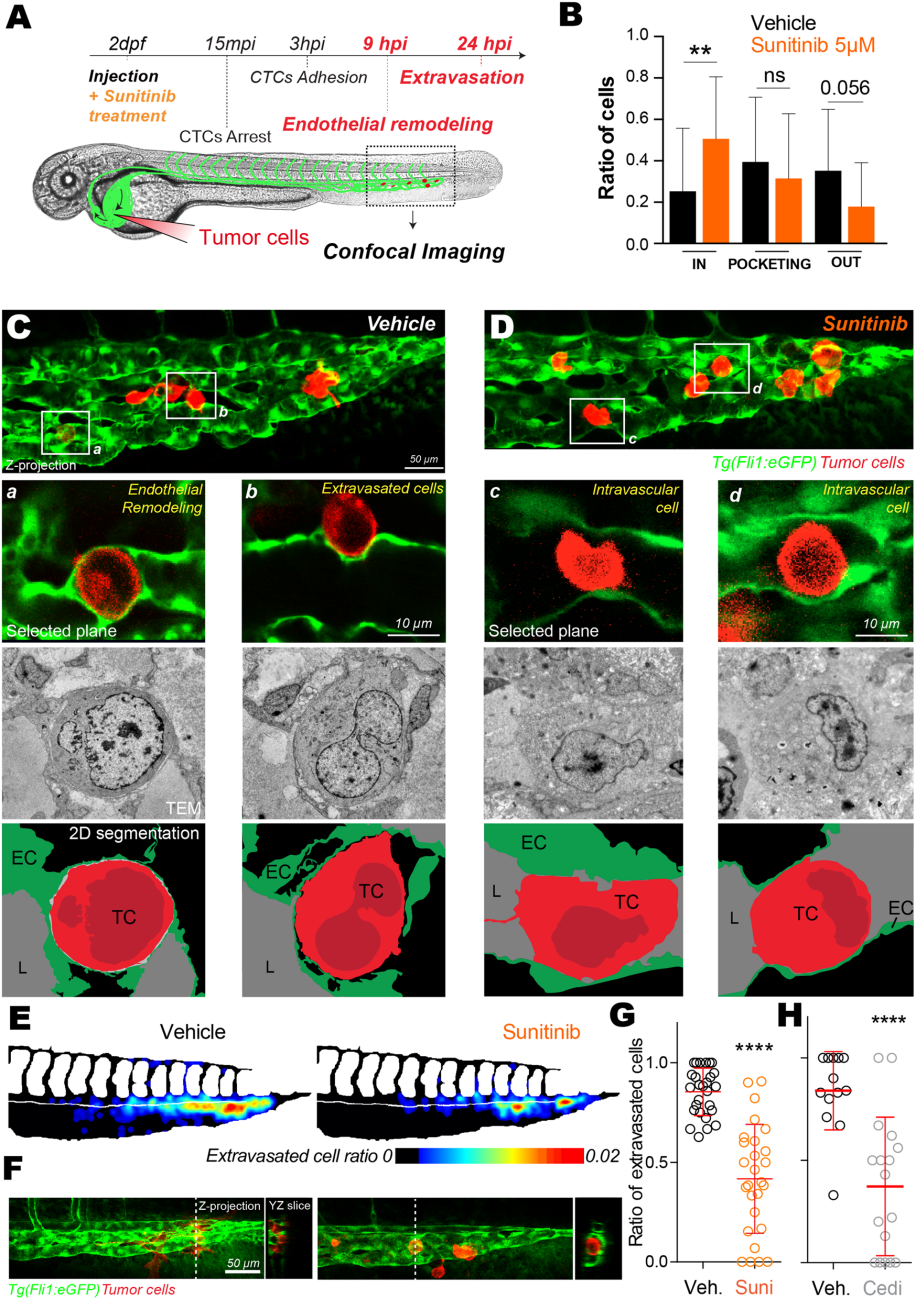
Inhibition of VEGFRs with sunitinib impacts extravasation by endothelial remodeling. **(A**) Experimental setup used: zebrafish are imaged at 9 hpi & 24 hpi.** (B)** Quantification of intravascular, remodeling and extravasated cells 9 hpi. N cells: vehicle = 134, sunitinib = 155. N embryos: vehicle = 23, sunitinib = 25, Kruskal–Wallis test followed by Dunn's multiple Comparison test. (**C,D)** Representative image of the caudal plexus by confocal intravital imaging (upper panel), corresponding correlative light and electron microscopy imaging (middle panel) and reconstructed segmented images (lower panel). In vehicle treated embryos, tumor cells of interest are *a* and *b* (white squares on (**C)**). In sunitinib treated embryos, tumor cells of interest are *c* and *d* (white squares on (**D)**). *TC* tumor cell, *EC* endothelial cell, *L* lumen. (**E)** The heatmaps show the quantification and location of extravascular CTCs at 24 hpi in the caudal plexus treated with vehicle or 2 µM of sunitinib. (**F)** Representative images and orthoslice at 24 hpi. (**G)** Quantification of extravasated cells ratio at 24 hpi treated with vehicle or 2 µM of sunitinib. N cells: vehicle = 588, sunitinib = 441. N embryos: vehicle = 27, sunitinib = 27, Mann Whitney test. (**H)** Quantification of extravasated cells ratio at 24 hpi treated with vehicle or 0.1 µM of cediranib. N cells: vehicle = 134, cediranib = 143. N embryos: vehicle = 13, cediranib = 17, Mann Whitney test.

We next investigated the role of VEGFR signaling during the latter steps of endothelial remodeling (Fig. 4A). We thus performed 3D confocal intravital imaging at 9 hpi where we previously demonstrated that most of extravasating cells are enwrapped by the endothelium, and thus engaged in the pro-extravasation endothelial remodeling process, or extravasated^9^. Inhibition of VEGFRs significantly impairs extravasation of tumor cells at 9hpi (Fig. 4B-D). More specifically, while a majority of the tumor cells remained intravascular or in the process of being pocketed upon VEGFR inhibition, ~ 80% of cells were either fully pocketed or extravasated in control embryos (Fig. 4B). To confirm these quantitative results and further investigate endothelial remodeling at high-resolution, we set out a correlative light and electron microscopy (CLEM) experiment on representative embryos in both conditions (Fig. 4C,D). CLEM analysis of embryos treated with vehicle immediately after cells injection into the vasculature allowed us to highlight cells that are either in the process of extravasation, through endothelial remodeling, or already fully extravasated and seemingly in contact with the perivascular environment (Fig. 4C). Cells undergoing active endothelial remodeling were fully enwrapped with a layer of endothelium observed all around the extravasating CTC (Fig. 4Ca). We also observed that extravasated cells were present in the perivascular niche and still in close contact with endothelial cells (Fig. 4Cb). When tracking tumor cells in sunitinib-treated embryos, we observed that they remained intravascular with no obvious ultrastructural protrusions that would suggest endothelial remodeling initiation (Fig. 4D). Although these cells were in close contact with the endothelium, we could not observe any protrusions from endothelial cells in close proximity suggesting that endothelial remodeling was either impaired or endothelial migration around the arrested CTCs was delayed (Fig. 4D).

We next investigated the effect of sunitinib treatment on the efficiency of extravasation and the formation of micrometastases. We previously demonstrated that the majority of tumor cells were extravasated at 24 hpi^9^. We thus quantified the ratio of extravasated tumor cells at 24 hpi in embryos that had been treated with vehicle or sunitinib over the whole course of the experiment. We confirmed that treating embryos for 24 h with 5 µM of sunitinib led to severe defects on the vasculature confirming sunitinib efficiency (data not shown). To rule out any deleterious effects on the vascular architecture that could result from high-dose sunitinib, we thus employed sunitinib at low dose (2 µM) where only mild perturbations to vascular anatomy could be observed after 24 h of treatment. We observed a 8–9% increase in ISV spacing which we considered as a mild effect compared to a 24 h treatment with 5 µM of sunitinib which fully abolishes ISVs (data not shown). We previously demonstrated that endothelial remodeling does not occur in ISVs, and most of the tumor cells will stop in the arterio-venous junction^9^. Thus this mild effect does not affect endothelial remodeling since the hotspot for extravasation is not affected. We did not measure any significant difference in blood flow speed nor in red blood cells counts (Fig S2A,B). Interestingly, in these conditions, the number of extravascular tumor cells was significantly decreased in the caudal plexus region upon VEGFR inhibition (Fig. 4E,F, S2C). In details, we measured a ~ twofold decrease in extravasation efficiency in sunitinib-treated embryos (Fig. 4G) with more CTC staying intravascular compared to vehicle-treated embryos (Fig. 4F). While this phenotype and drug effect raise very exciting avenues in the treatment of metastasis, sunitinib is known to affect other tyrosine kinase receptors in addition to VEGFR (PDGFRs, c-Kit, RET, G-CSF-R, M-CSF-R, FLT3…)^37^. We thus sought to demonstrate the specific involvement of VEGFR using cediranib, a tyrosine kinase inhibitor with higher specificity for VEGFRs and more specifically toward KDR/VEGFR2^38^. Cediranib was previously used in zebrafish to inhibit angiogenesis of syngeneic ERMS tumors^39^. Using a similar experimental strategy, we treated embryos with 0.1 µM of cediranib following tumor cells injection. We observed that extravasation was reduced in cediranib-treated embryos to the same extent as sunitinib-treated embryos (Fig. 4H). To rule out any side effect of cediranib on the vasculature, we confirmed that cediranib did not alter the vascular architecture nor blood flow velocity at working concentration (Fig S3A,B). Interestingly again, the treatment regime that we used was potent in inhibiting CTC extravasation by targeting VEGFR-dependent intraluminal remodeling without causing major vascular defects. Taken together, these in vivo experiments in a validated experimental metastasis assay demonstrate that blood flow dependent indoctrination of endothelial cells requires VEGFR2 and the VEGFR signaling pathway to favor extravasation of tumor cells in zebrafish embryos.

## Discussion

CTC extravasation is an essential step preceding the rate-limiting organ colonization and the foundation of secondary tumor foci^17^. Yet, the cellular dynamics and molecular mechanisms of CTC extravasation remain incompletely understood. Extravasation of tumor cells is a rare event at the scale of an entire organism and full understanding requires resolutive imaging technologies applied to relevant experimental metastasis models. Based on this, we previously discovered that extravasation occurs mainly through an active endothelial-dependent process that we named endothelial remodeling^9^. Our findings were recently extended to human melanoma and cervix cancer lines^19^, suggesting that it is not a cell line specific mechanism. Incidentally, a similar process had also been linked to extravasation of stem cells^40^ and is at play during the important step of blood clot removal, through angiophagy^21, 41^. While recent work demonstrated that endothelial cell death through necroptosis is important for tumor cell extravasation^42^, earlier work had also suggested endothelialization of tumor cells during metastasis^43,44^. In addition, while such extravasation of tumor cells using endothelial remodeling remains to be documented and functionally tested in classical metastatic organs (lungs, liver, etc.…), we and others had shown that it is very likely an important process in the context of mouse brain metastasis^9,20^. Altogether, this demonstrates that the endothelial wall is a key determinant of the metastatic success. Yet, the underlying molecular triggers and mechanisms favoring plasticity of the endothelium during extravasation remain, at this stage, poorly understood.

Using a flow-tuning approach in the zebrafish embryo and in vitro microfluidics, we demonstrated that endothelial remodeling is driven and tuned by hemodynamic cues from the blood flow^9^. Here, using a combination of in vitro microfluidics and transcriptomics, but also of intravital imaging and ultrastructural analysis in the zebrafish embryo, we demonstrate that endothelial remodeling around arrested tumor cell is driven, in part, by VEGFR signaling downstream of hemodynamic cues. This is further supported by the observation that endothelial remodeling can be impaired using VEGFR inhibitors initially developed to prevent tumor neo-angiogenesis and used as adjuvant to chemotherapies. We thus demonstrate that intravascular cues that are at play during metastasis are capable of indoctrinating the endothelial wall through a classical VEGFR pathway to stimulate metastatic extravasation of CTCs.

Using transcriptomic analysis, we first demonstrate that permissive flow profiles favor the activation of molecular programs that are linked to angiogenesis, as previously described during developmental angiogenesis^30,45–47^. Within this molecular program, VEGF receptors, and more specifically KDR (VEGFR2), appear significantly upregulated in response to flow. Endothelial cells have developed several strategies to sense flow which include the cilium, the glycocalyx, mechano-sensitive ions channels, G protein coupled receptors, caveolae, adhesion receptors and VEGFRs^48,49^. Thus, mechanisms that are relevant for developmental angiogenesis are also likely to be essential for pathological vessel remodeling, including during metastatic extravasation.

With the idea to further validate a molecular node that controls extravasation of tumor cells through endothelial remodeling, we decided to employ sunitinib and cediranib, as potent endothelial remodeling inhibitors. These anti-VEGFR treatments successfully inhibit the extravasation of tumor cells both in an in vitro microfluidic setup and in the zebrafish embryos. Our data argue that the VEGFR2 pathway is a major actor of the flow-dependent endothelial remodeling, driving extravasation as we described previously^9^. Whether VEGFR, and more specifically the mechanosensory VEGFR2 and 3, are direct flow mechano-transducers, are acting together with other mechanosensory complexes or are activated downstream of other flow sensors remains to be elucidated^48^. The VEGFR canonical activation is complex, due to partial ligand specificity and crosstalk between VEGF A, B, C, D or PIGF ligands and VEGFR1, R2 or R3 receptors and the heterodimerization between the VEGFRs^29, 50^. Interestingly, non-canonical (ligand-independent) VEGFR activation has been described in the context of mechano-transduction^31,51–53^. Blood flow changes that occur at the apical side of the endothelial cell, when CTC arrest, might be sufficient to activate VEGFR kinase cascade and transduce the signal into the endothelial cells^54,55^. Alternatively, indirect forces that result from shear forces on the arrested CTC and that could be transmitted to the endothelial cell which is directly engaged through CTC-endothelium cell–cell adhesions on the luminal side are likely to favor certain transcriptional programs^32,56^. Such a hypothesis is exciting and further work is needed to determine whether indirect flow forces on arrested tumor cells are likely to impact neighboring endothelial cells. Combination of biophysical tools (such as optical tweezing technologies to manipulate CTC and/or endothelial cells) with microfluidic approaches could be helpful in that regard^4,9,57^. In addition, there are obviously additional molecular programs that are likely to be involved during this endothelium-dependent process. Interestingly, the process of angiophagy is driven by MMP2/9 activity^21^ and could be involved in driving endothelial remodeling that highly depends on endothelial cell protrusions. In our case, however, expression levels of MMP 2 and 9 are only mildly affected (data not shown). Furthermore, more work is needed to demonstrate whether endothelial remodeling is a universal process used by CTCs to extravasate, or this is limited to certain vascular and hemodynamic conditions or to specific organs. Indeed, the latter likely depends on the organ of metastasis. Although the vasculature of the early zebrafish embryo differs from mature vessels of other relevant models such as mice, we had shown that endothelial remodeling is also initiated during metastatic extravasation of tumor cells in the mouse brain^9^. In that case, VEGFR or other signaling pathways could control endothelial remodeling and metastatic extravasation. More work is required to determine whether endothelial remodeling occurs in distinct vascular environments, within distinct metastatic organs, and whether they use common or alternative signaling pathways.

Sunitinib is a tyrosine-kinase receptor inhibitor, mainly targeting VEGFR, PDGFR and c-KIT receptors that was first identified in 1999^58^. It is currently clinically used for several cancer treatments including renal or gastrointestinal cancer^59^. Several clinical trials are being conducted to block vascular development around well-established tumors aiming at comparing the effects of sunitinib and cediranib with similar drugs such as pazopanib, another VEGFR/PDFGR/cKit Inhibitor^60,61^. Being with bevacuzimab and to a lesser extent imatinib, the only anti-VEGF therapies clinically approved and despite positive results in its ability to impair tumor growth and cancer progression^62^, the clinical impact of sunitinib is currently questioned as contradictory results have been obtained in the context of tumor metastasis. At this stage, although sunitinib displays detrimental properties that impair its usage in this context, we believe that its powerful inhibition of the VEGFR pathway demonstrates that metastatic extravasation of tumor cells is likely to occur through VEGFR-dependent endothelial remodeling. These results are further strengthened by the observation that cediranib, a VEGFR-specific tyrosine kinase inhibitor phenocopies sunitinib effects on CTC extravasation. In line with our results, a recent study used zebrafish model to predict the clinical efficiency of bevacizumab, another anti-angiogenic therapeutic agent, in anti-metastatic response^63^. Inhibition of vascular endothelial growth factor-A (VEGF-A) also induced long-term dormancy of lung cancer micrometastases by preventing angiogenic growth to macrometastases in a mouse model of brain metastasis^6^. Flow-independent biomechanical cues might also be elemental to the endothelial response during metastatic progression. It was recently demonstrated that stiffness reduction in liver metastasis induced a higher therapeutic response to bevacizumab^64^, similarly to our observations pointing at a crosstalk between VEGFR-mediated endothelial response and external biomechanical cues. More work is needed to identify additional signaling pathways, and more specific molecules, to efficiently counteract this new mechanism of metastatic extravasation.

Altogether, our data argue that the endothelial remodeling leading to extravasation of metastatic cells can be chemically inhibited in vivo. Sunitinib appears as a promising drug to specifically target tumor cells extravasation whose efficiency should be carefully assessed in mouse experimental metastasis models.

## Material and methods

### Cell lines

D2A1 were provided by Robert A. Weinberg (MIT). Cells stably expressing LifeAct-TdTomato were obtained using lentiviral transfection. Cells were grown as previously described^65^, in DMEM with 4.5 g/l glucose (Dutscher) supplemented with 5% FBS, 5% NBCS, 1% NEAA and 1% penicillin–streptomycin (Gibco). Human Umbilical Vein Endothelial Cells (HUVEC) (PromoCell) were grown in ECGM (PromoCell) supplemented with supplemental mix (PromoCell C-39215) and 1% penicillin–streptomycin (Gibco). To maximize the reproducibility of our experiments, we always used these cells at 4th passage in the microfluidic channels.

### Zebrafish handling and sunitinib/cediranib treatment

We used the following zebrafish (*Danio rerio*) lines *Tg(fli1a:EGFP)*^66^*, Tg(kdrl:EGFP; gata1:DsRed)*^67^. We generated the following lines by crossing and selecting double positive embryos: *Tg(fli1a:EGFP; gata1:DsRed)*^66,68^ and *Tg(CD41:EGFP; gata1:DsRed)*^68,69^. Embryos were maintained in Danieau 0.3X medium (17.4 mM NaCl, 0.2 mM KCl, 0.1 mM MgSO_4_, 0.2 mM Ca(NO_3_)_2_) buffered with HEPES 0.15 mM (pH  7.6), supplemented with 200 µM of 1-Phenyl-2-thiourea (Sigma-Aldrich) to inhibit the melanogenesis, as previously described ^70^. Sunitinib (Sigma-Aldrich) was added in the breeding water of the embryos directly after injection of tumor cells at the concentration of 2 µM or 5 µM vs vehicle (DMSO). Cediranib (BioVision) was added in the breeding water of the embryos directly after injection of tumor cells at the concentration of 0.1 µM vs vehicle (DMSO).

### Control experiments for sunitinib & cediranib effect on zebrafish embryos

Sunitinib and cediranib treatment were tested for their potential impact on vascular architecture and/or on blood flow pattern in the caudal region. After 3 or 24 h of treatment (sunitinib, cediranib or vehicle), we performed confocal microscopy using an inverted TCS SP5 confocal microscope with a 20× /0.75 (Leica) and manually analyzed the architectures of landmark vessels. In the plexus, the conservation of the distance between intersegmental vessels and the number of vascular branching in the caudal veins was quantified using ImageJ software. Also, we performed fast recording using the resonant scanner at a speed of 27 fps in *Tg(kdrl:EGFP; gata1:DsRed)* or *Tg(CD41:EGFP; gata1:DsRed)* embryos. We measured the mean flow velocity using the TrackMate plugin^71^ in Fiji 1.52n (https://fiji.sc/).

To measure red blood cells counts, the number of red blood cells in individual movies was counted in the dorsal aorta of embryos and normalized to the length of the region of interest which had been considered.

### Intravascular injection and imaging of CTCs in the zebrafish embryo

48 h post-fertilization (hpf) *Tg(Fli1a:EGFP)* embryos were mounted in 0.8% low melting point agarose pad containing 650 µM of tricain (ethyl-3-aminobenzoate-methanesulfonate) to immobilize the embryos. D2A1 LifeAct-TdTomato cells were injected with a Nanoject microinjector 2 (Drummond) and microforged glass capillaries (25 to 30 µm inner diameter) filled with mineral oil (Sigma). 18 nL of a cell suspension at 100.10^6^ cells per ml were injected in the duct of Cuvier of the embryos under the M205 FA stereomicroscope (Leica).

For caudal plexus, confocal imaging was performed with an inverted TCS SP5 confocal microscope with a 20× /0.75 (Leica). The caudal plexus region (around 50 µm width) was imaged with a z-step of less than 1.5 µm for at least 20 embryos per conditions from 3 independent experiments. Cell number and situations was manually characterized (Intravascular, ongoing endothelial remodeling/pocketing, extravascular) using z-projections and orthogonal views in ImageJ.

Correlative Light and Electron Microscopy was performed to describe ultrastructural characteristics of CTCs and the endothelium in the zebrafish embryo. Chosen embryos of both condition (Vehicle and Sunitinib treated) were imaged using confocal microscopy between 3 to 4 hpi. Just after imaging, they were chemically fixed and processed for EM (see dedicated section “EM preparation”).

### EM preparation and correlation between light and electron microscopy

The samples (fish tails after confocal microscopy) have been post fixed in a solution of 2,5% glutaraldehyde and 2% paraformaldehyde in 0.1 M Cacodylate buffer at 4 °C overnight. Samples were rinsed in 0.1 M Cacodylate buffer for 2 × 5 min and post-fixed using 1% OsO4 in 0.1 M Cacodylate buffer, for 1 h at room temperature. Then, samples were rinsed for 3 × 5 min in 0.1 M Cacodylate buffer, followed by washing 2 × 5 min in pure water. Samples were secondary post-fixed with 4% water solution of uranyl acetate for 1 h at room temperature. Followed by 5 min wash in pure water, the samples were stepwise dehydrated in Ethanol (50% 3 × 5 min, 70% 3 × 10 min, 90% 3 × 10 min and 100% 3 × 10 min) and infiltrated in a graded series of Epon (Ethanol abs/Epon 3/1, 1/1, 1/3, each 30 min). Samples were left in absolute Epon (EmBed812—EMS) overnight. Then, samples were placed in a fresh absolute Epon for 1 h and polymerized (flat embedded) at 60 °C for 24-48 h. Once polymerized, most surrounding Epon was cut off using razorblade and samples were mounted on empty Epon blocks (samples flat at the surface of the blocks) and left at 60 °C for 24 h-48 h.

To do the correlation in 3d, semi thin sections (500 nm) were obtained using glass knife in ultramicrotome LEICA UCT. Sections were placed on slide, stained with 1% borax Toluidine blue solution and checked out in the optical microscope. Anatomical landmarks were used to retrieve the ROI (dorsal aorta, tumor cells…).

Ultrathin sections (100 nm) were serially sectioned using ultramicrotome (Leica Ultracut UCT), collected on formvar-coated slot grids and stained with 4% water solution of uranyl acetate for 5 min and lead citrate for 3 min. Ultra-thin sections were imaged with a CM120 transmission electron microscope (Philips Biotwin) operating at 120 kV. Images were recorded with Veleta 2 k  ×  2 k (Olympus-SIS) camera using iTEM 5.0 software. The ROI was approached by progressive serial sectioning of the samples. Multiple sections were further processed and acquired.

### Zebrafish heatmaps

The heatmaps were generated as previously described^36^ using ImageJ 1.52n (https://imagej.nih.gov/ij/index.html) and MATLAB R2016b (MathWorks, https://www.mathworks.com/) softwares.

### Microfluidic experiments

For endothelial remodeling experiments in vitro, two µ-slides I^0.4^ Luer (IBIDI) coated with fibronectin from bovine plasma at 10 µg/ml (Sigma F-1141) were used in parallel for each experiment. HUVEC cells were seeded at 100 000 cells per channel. Medium was changed twice a day for 2 or 3 days, before perfusing the channels under a flow of 400 µm/s using REGLO Digital MS-2/12 peristaltic pump (Ismatec) and Tygon tubbing (IDEX). Sunitinib treatment was added at a concentration of 0.1 µM in flow. At confluence, D2A1 LifeAct-TdTomato cells were added at a concentration of 200,000 cells/ml for 10 min. Then, floating tumor cells were washed using fresh medium and the channels were incubated for 16 h with flow. Position of the tumor cells and presence of endothelial remodeling around tumor cells relative to the HUVEC monolayer were determined using the piezo stage of the confocal microscope after fixation and Immunofluorescent staining (*see *next section).

### Antibodies

Mouse anti-human CD31 (PECAM) monoclonal antibody (MEM-5) was purchased from Thermo, mouse anti-human FLT1 (ab9540) and rat anti-mouse FLT4 (ab51874) monoclonal antibodies were purchased from AbCam, rabbit anti-KDR monoclonal antibody (D5B1) was purchased from Cell Signaling and α-tubulin (DM1A) was purchased from Millipore. Fluorescently labelled secondary antibodies were purchased from Invitrogen: goat anti-mouse/rat/rabbit coupled with Alexa Fluor 488, Cy3, Alexa 555, Cy5 or Alexa 647. HRP-conjugated secondary antibodies were purchased form Jackson Immunoresearch.

### Immunofluorescent staining in the microfluidic channels

Cells were fixed using 4% PFA (Electronic Microscopy Sciences), permeabilized with 0.2% Triton-X100 (Sigma) and quenched with 2 mg/ml NaBH_4_ (Sigma) 10 min at room temperature before using the following primary antibodies. Cells were incubated 1 h at room temperature on a tilting stage and washed with PBS. Cells were incubated with secondary antibodies 30 min at room temperature on a tilting stage and protected from light. Cells were mounted using Vectashield (Vector Laboratories).

### Western blotting

Channels were seeded simultaneously with 100,000 cells 4 days prior to experiment. Extracts corresponding to similar cell numbers were loaded on 4–20% polyacrylamide gels (Biorad) and run under denaturing conditions. Primary antibodies were incubated 2 h at room temperature. HRP-conjugated secondary antibodies were incubated 1 h at room temperature. Data were acquired using with ECL (GE Healthcare) and the ChemiDoc XRS (Biorad). Intensities were normalized over α-tubulin levels.

### RNA sequencing

3 independent couples (flow and no flow) of HUVEC samples were isolated from µ-slides I^0,4^ Luer (ibidi) using Tri-reagent (MRC) 100 µl of Tri-reagent was added directly in one side of the channel and aspire in the other side 5 times. This was followed by chloroform extraction and alcohol washing. Total cDNA was obtained using Thermo Fisher kit (SuperScript VILO Master mix).

RNA integrity was assessed with the Agilent total RNA Pico Kit on a 2100 Bioanalyzer instrument (Agilent Technologies, Paolo Alto, USA). The construction of libraries was done with the "SMARTer Stranded Total RNA-Seq Kit v2—Pico Input Mammalian" (TaKaRa Bio USA) with a final multiplexing of 12 libraries according to the manufacturer's instructions. The library pool was denatured according to the Illumina protocol "Denature and Dilute Libraries Guide" and then deposited at a concentration of 1.3 pM to be sequenced on the NextSeq 500 (Illumina).

The transcriptome data set, composed of sequencing reads, was generated by an Illumina NextSeq instrument. The objective is to identify genes that are differentially expressed between two experimental conditions: *flow* and *no flow*. First, the data were mapped to the human genome/transcriptome (hg19) using the HISAT2 software v2.1.0 (http://daehwankimlab.github.io/hisat2/) ^72,73^, a fast and sensitive alignment program. The total reads mapped were finally available in BAM format for raw read counts extraction. Next, read counts were found by the htseq-count tool of the Python package HTSeq^74^ with default parameters to generate an abundant matrix. Then, differential analyses were performed by the DESEQ2^75^ package of the Bioconductor framework. Up-regulated and down-regulated genes were selected based on the adjusted p-value cutoff 10%. Finally, Gene Ontology Consortium (http://www.geneontology.org/) platform was used for data analysis and heatmaps creation. The heatmaps were formatted using IGOR pro 8 software (https://www.wavemetrics.com/).

### qPCR validation

3 to 7 independent couples (flow and no flow) of HUVEC samples total RNAs were isolated from µ-slides I^0,4^ Luer (ibidi) using Tri-reagent (MRC) followed by chloroform extraction and alcohol precipitation. Total cDNA was obtained using ThermoFisher kit (SuperScript VILO Master mix). RT qPCR reactions were made using either TaqMan master mix (ThermoFisher—4444557) or SYBR green master mix (Thermo Fisher—A25742) in an Applied Biosystem qPCR machine (QuantStudio 3—Thermo Fisher). See next table of qPCR primer sequences for Flt1, Kdr, Flt4, Notch1, Lama4 (lab designed). For human GAPDH, a commercial TaqMan probe was used (Thermo Fisher—4333764F). Amplification results were normalized using GAPDH level and double ΔcT method^76^.

**Table:** 

Targets	Primer sequences
h Flt1 fwd	CCA GCA GCG AAA GCT TTG CG
h Flt1 rev	CTC CTT GTA GAA ACC GTC AG
h Kdr fwd	ATG ACA TTT TGA TCA TGG AGC
h Kdr rev	CCC AGA TGC CGT GCA TGA G
h Flt4 fwr	TGC AAG AGG AAG AGG AGG TCT
h Flt4 rev	CAG GCT TGG CGG GCT GTC C
h Notch1 fwd	CAG GAC GGC TGC GGC TCC TAC
h Notch1 rev	CCG CCG TTC TTG CAG GGC GAG
h Lama4 fwr	ACC TCC TCA ATC AAG CCA GA
h Lama4 rev	TCA GCC ACT GCT TCA TCA CT

### RT-qPCR in FACS-sorted zebrafish endothelium

Embryos were euthanized using tricaine overdose collected in 1.5 ml Eppendorf tubes (30 embryos per tube). Embryos are washed twice with 1 ml of 1× PBS and 500 µl of the dissociation mix (collagenase 4 mg/ml in 0.25% trypsin–EDTA pre-heated at 30 ˚C) was added and each tube was incubated at 30 °C. Embryos were mechanically dissociated and homogenized using harsh pipetting until tissues were no longer visible. 800 µl of DMEM-10% FBS was added to the tubes to stop the dissociation. Tubes were mixed and centrifuged at 700*g* for 5 min. Pellets were resuspended in 1 ml of 1× PBS and centrifuged at 700*g* for 5 min. The pellet was resuspended in 500 µl of cell-sorting buffer (5 mM EDTA, 25 mM HEPES, 2% FCS in 1× PBS). Samples were sorted through 70 µm nylon-mesh and collected in a new Eppendorf tube for FACS sorting. GFP + cells were sorted using an Aria II SORP FACS cell sorter (BD Bioscience). Total RNAs were isolated from 200,000 GFP + FACS-sorted cell population using Tri-reagent (MRC) followed by chloroform extraction and alcohol precipitation. Total cDNA was obtained using High-Capacity cDNA Reverse Transcription Kit (Thermo Fischer—4368814). RT-qPCR reactions were made using either TaqMan master mix (Thermo Fisher—4444557) or SYBR green master mix (Thermo Fisher—A25742) in an Applied Biosystem qPCR machine (QuantStudio 3—Thermo Fisher). Commercial TaqMan probes were used KDR (Thermo Fisher—Dr03116261_m1) and KDRL (Thermo Fisher—Dr03432904_m1). Zebrafish GAPDH primers forward: tcagtccactcacaccaagtg, reverse: cgaccgaatccgttaatacc. Amplification results were normalized using GAPDH level and double ΔcT method^76^.

### Statistical analysis

Statistical analysis was performed using the GraphPad Prism program version 5.04 (https://www.graphpad.com/). The Shapiro–Wilk normality test was used to confirm the normality of the data. For data following a Gaussian distribution, a Student unpaired two-tailed t test, with Welch's correction in case of unequal variances was used. For data not following a Gaussian distribution, the Mann–Whitney test was used. When more than 3 groups are compared, a Kruskal–Wallis test followed by Dunn's multiple comparison test was used. For qualitative data, the Fisher test was used. Illustrations of these statistical analyses are displayed as the mean + /− standard deviation (SD). p-values smaller than 0.05 were considered as significant. *, p < 0.05, **, p < 0.01, ***, p < 0.001, ****, p < 0.0001, ns = not significant.

## Supplementary Information

Supplementary Figures.
