# Author Correction: Impairing flow-mediated endothelial remodeling reduces extravasation of tumor cells

**DOI:** 10.1038/s41598-021-97172-z

**Published:** 2021-09-01

**Authors:** Gautier Follain, Naël Osmani, Valentin Gensbittel, Nandini Asokan, Annabel Larnicol, Luc Mercier, Maria Jesus Garcia‑Leon, Ignacio Busnelli, Angelique Pichot, Nicodème Paul, Raphaël Carapito, Seiamak Bahram, Olivier Lefebvre, Jacky G. Goetz

**Affiliations:** 1 Tumor Biomechanics, INSERM UMR_S1109, CRBS, 67000 Strasbourg, France; 2 Université de Strasbourg, 67000 Strasbourg, France; 3 Fédération de Médecine Translationnelle de Strasbourg (FMTS), 67000 Strasbourg, France; 4 Equipe Labellisée Ligue Contre le Cancer, Paris, France; 5 Present Address: Turku Bioscience Center, University of Turku, Åbo Akademi University, 20520 Turku, Finland; 6 Present Address: UMR 5297, Interdisciplinary Institute for Neurosciences, CNRS Université de Bordeaux, 33076 Bordeaux, France

Correction to: *Scientific Reports* 10.1038/s41598-021-92515-2, published online 23 June 2021

The original version of this Article contained an error in Figure 4 where panel (c), (d) and (f) were incorrectly displayed. The original Figure 4 and accompanying legend appear below.

**Figure 4:**
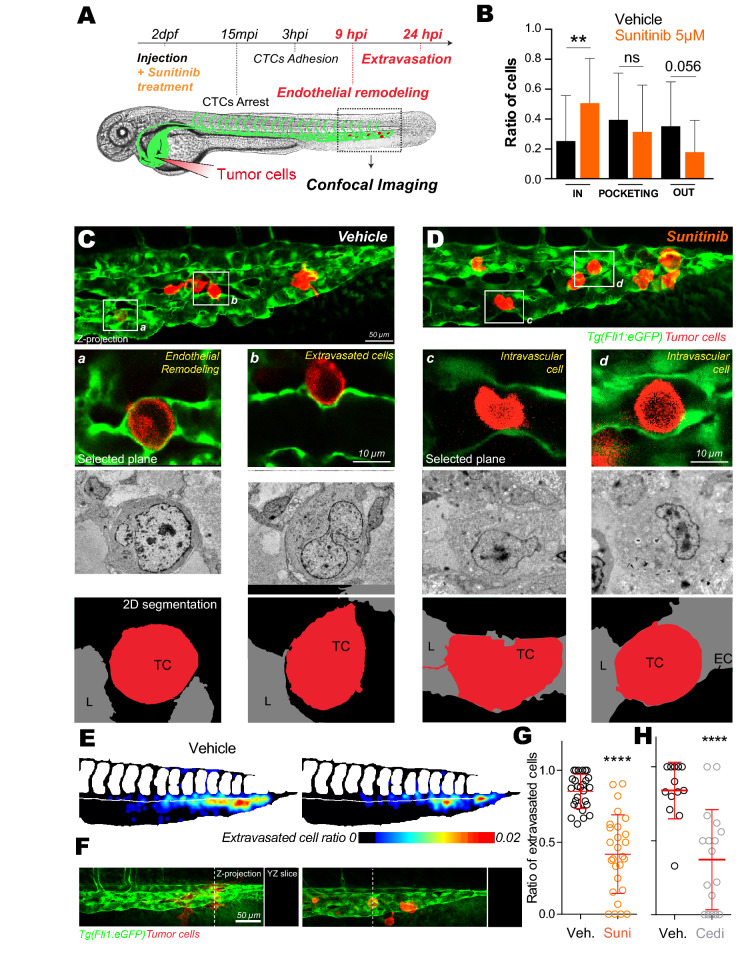
Inhibition of VEGFRs with sunitinib impacts extravasation by endothelial remodeling. **(A)** Experimental setup used: zebrafish are imaged at 9 hpi & 24 hpi. **(B)** Quantification of intravascular, remodeling and extravasated cells 9 hpi. N cells: vehicle = 134, sunitinib = 155. N embryos: vehicle = 23, sunitinib = 25, Kruskal–Wallis test followed by Dunn's multiple Comparison test. **(C,D)** Representative image of the caudal plexus by confocal intravital imaging (upper panel), corresponding correlative light and electron microscopy imaging (middle panel) and reconstructed segmented images (lower panel). In vehicle treated embryos, tumor cells of interest are *a* and *b* (white squares on **(C)**). In sunitinib treated embryos, tumor cells of interest are *c* and *d* (white squares on **(D)**). *TC* tumor cell, *EC* endothelial cell, *L* lumen. **(E)** The heatmaps show the quantification and location of extravascular CTCs at 24 hpi in the caudal plexus treated with vehicle or 2 µM of sunitinib. **(F)** Representative images and orthoslice at 24 hpi. **(G)** Quantification of extravasated cells ratio at 24 hpi treated with vehicle or 2 µM of sunitinib. N cells: vehicle = 588, sunitinib = 441. N embryos: vehicle = 27, sunitinib = 27, Mann Whitney test. **(H)** Quantification of extravasated cells ratio at 24 hpi treated with vehicle or 0.1 µM of cediranib. N cells: vehicle = 134, cediranib = 143. N embryos: vehicle = 13, cediranib = 17, Mann Whitney test.

The original Article has been corrected.

