# Supplementary material for: Impairing flow-mediated endothelial remodeling reduces extravasation of tumor cells
Source: Sci Rep. 2021 Jun 23;11:13144. doi: 10.1038/s41598-021-92515-2 (PMC8222393; doi:10.1038/s41598-021-92515-2)
Supplement: Supplementary file 1 — Supplementary Figures. [file 41598_2021_92515_MOESM1_ESM.pdf]

SUPPLEMENTAL FIGURE LEGENDS

Figure S1: Gene expression in HUVEC cells treated with or without flow. A – Fold changes heatmap, based on GO class: Cell Cycle, showing significantly impacted genes in flow condition compared to no flow. Each lane represents a gene. Gene names have been alternatively written on left and right of the graph to ease reading. **B** – RT-qPCR data confirming RNA sequencing results. Graphic shows the comparison between RT-qPCR and RNAseq. Graph shows biological replicates, Mann Whitney test. **C** – Fold change of expression level (RNAseq) of the major sunitinib targets in flow vs no flow condition (numbers are adjusted p-values). **D** – RNA expression of KDR and KDRL in *Tg(fli1a:EGFP)* embryos treated with vehicle or lidocaine for 14h prior to tissue dissociation and FACS sorting or GFP+ cells. N embryos : EtOH1=42, EtOH2=43, Lido1=42, Lido2=43 (upper panel). Quantification of pacemaker activity. N embryos : EtOH=12, Lido=11 (bottom panel), Mann Whitney test.

Figure S2: Inhibition of VEGFRs with sunitinib reduces extravasation in the caudal plexus. A – Quantification of the effect of sunitinib on vessel architecture after 24h of treatment, N embryos: vehicle = 27, sunitinib 5 µM = 27, Mann Whitney test. Quantification of the effect of sunitinib on blood flow after 24h of treatment, N embryos: vehicle = 12, sunitinib 2 µM = 15, Mann Whitney test. **B** – Quantification of the effect of sunitinib on red blood cell counts after 3h or 24h of treatment with 5 and 2 µM respectively, N embryos: vehicle 3h = 8, sunitinib 5µM 3h = 10, vehicle 24h = 9, sunitinib 2µM 24h = 11, unpaired t test. The **C** – The heatmaps show the quantification and location of CTCs at 24 hpi in the caudal plexus after 24h of treatment with vehicle or 2 µM of sunitinib. N embryos: vehicle = 13, sunitinib 2 µM = 18, unpaired t test.

Figure S3: Inhibition of VEGFRs with cediranib reduces extravasation in the caudal plexus. A – Quantification of the effect of cediranib on vessel architecture after 24h of treatment, N embryos: vehicle = 10, cediranib 0.1 µM = 15, unpaired t test. **B** – Quantification of the effect of cediranib on blood flow after 24h of treatment, N embryos: vehicle = 12, cediranib 0.1 µM = 14, unpaired t test.

Figure S4: Gene ontology analysis for *in vitro* RNAseq data. Results from global Gene Ontology (GO) analysis on 'biological process' and 'cellular process', showing most significantly impacted GO classes.

Figure S5: Uncropped western blot membranes. 5 independent experiments were blotted simultaneously. The protein ladder has been superposed to uncropped membrane acquisitions to display molecular weights.

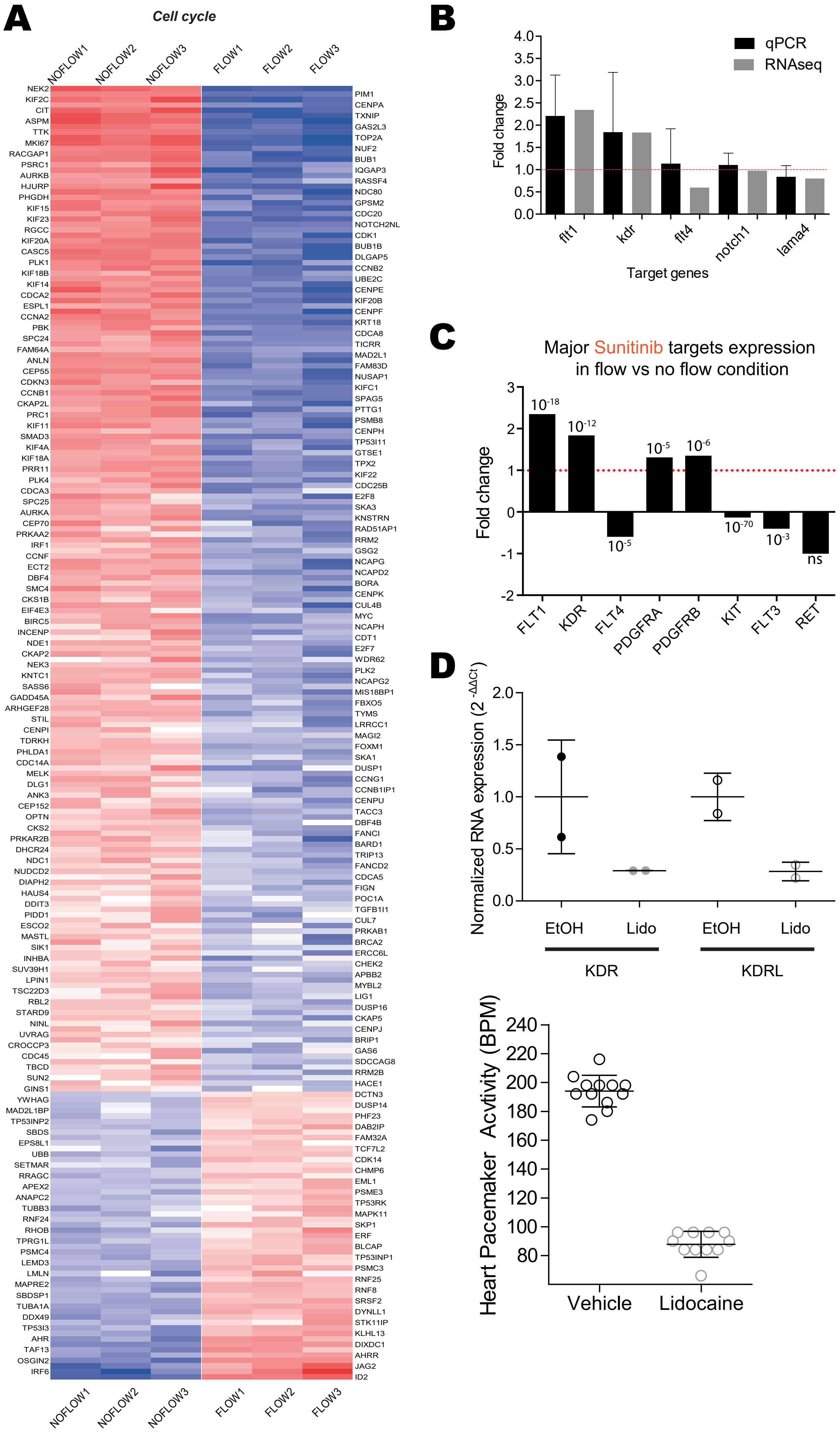

Supplementary Figure 1 - Follain_Osmani et al.

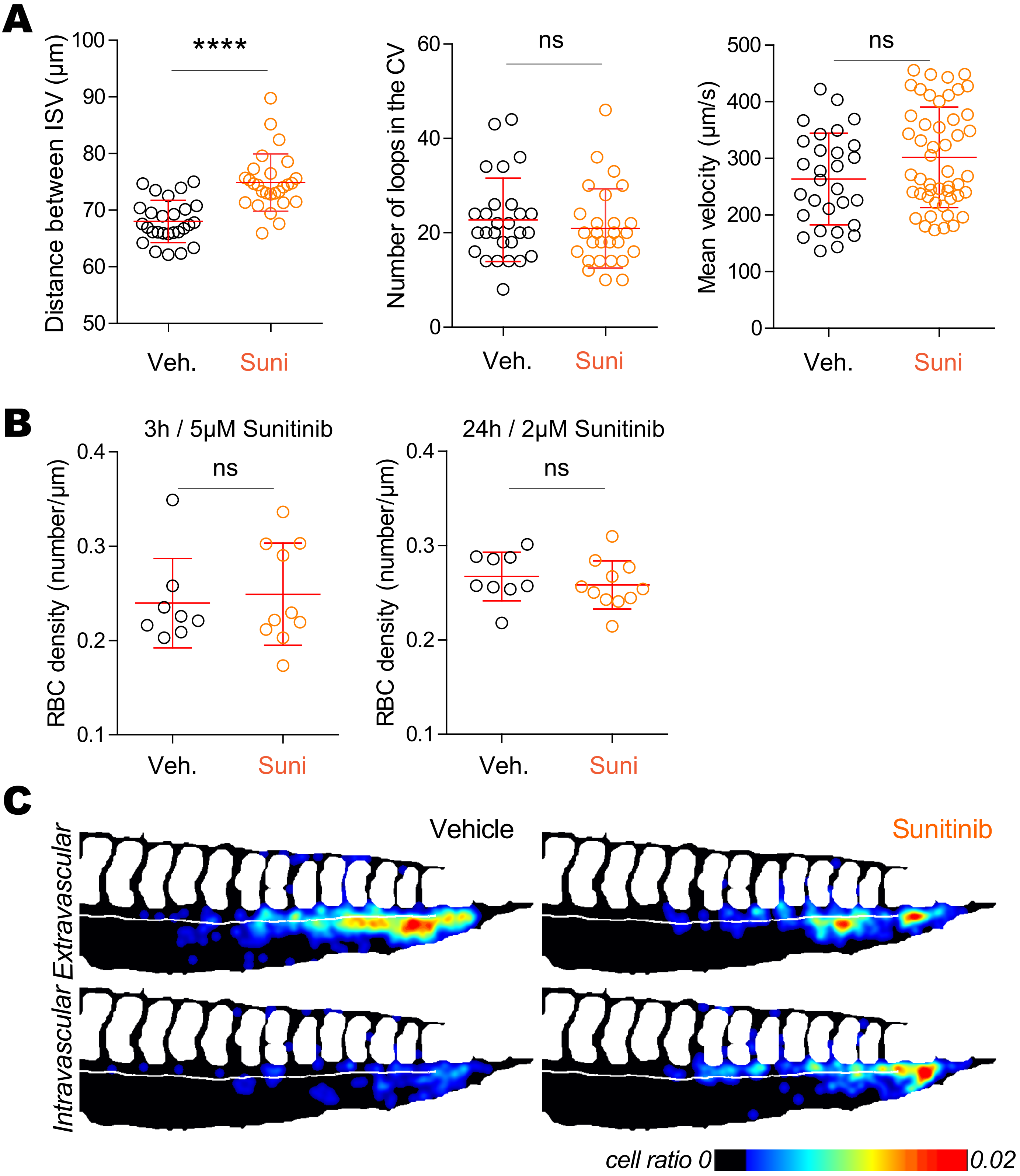

Supplementary Figure 2 - Follain_Osmani et al.

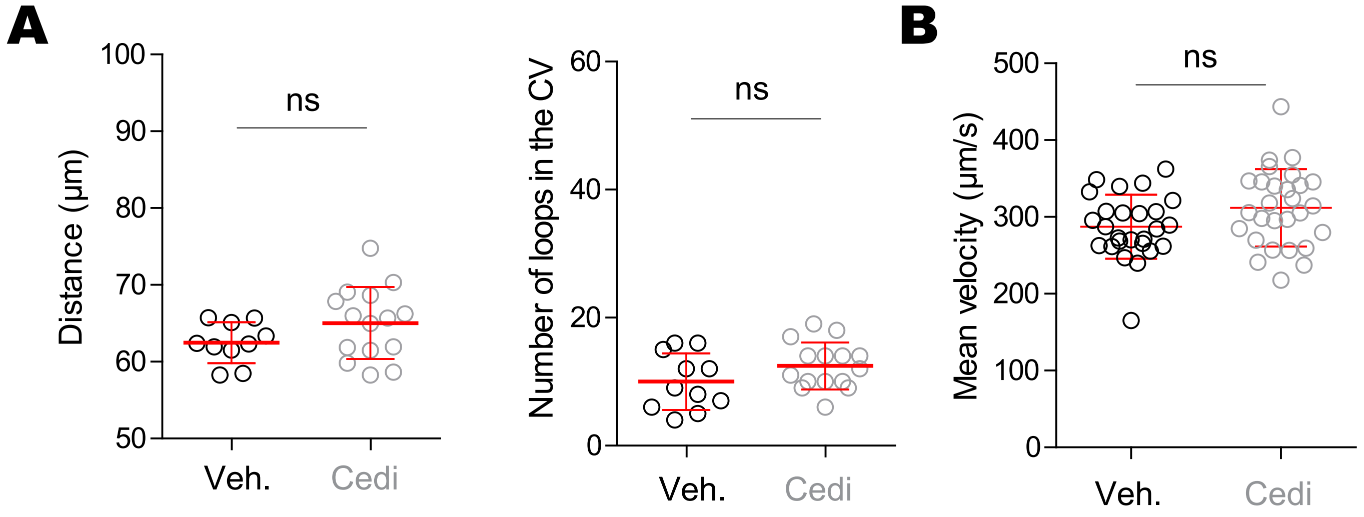

Supplementary Figure 3 - Follain_Osmani et al.

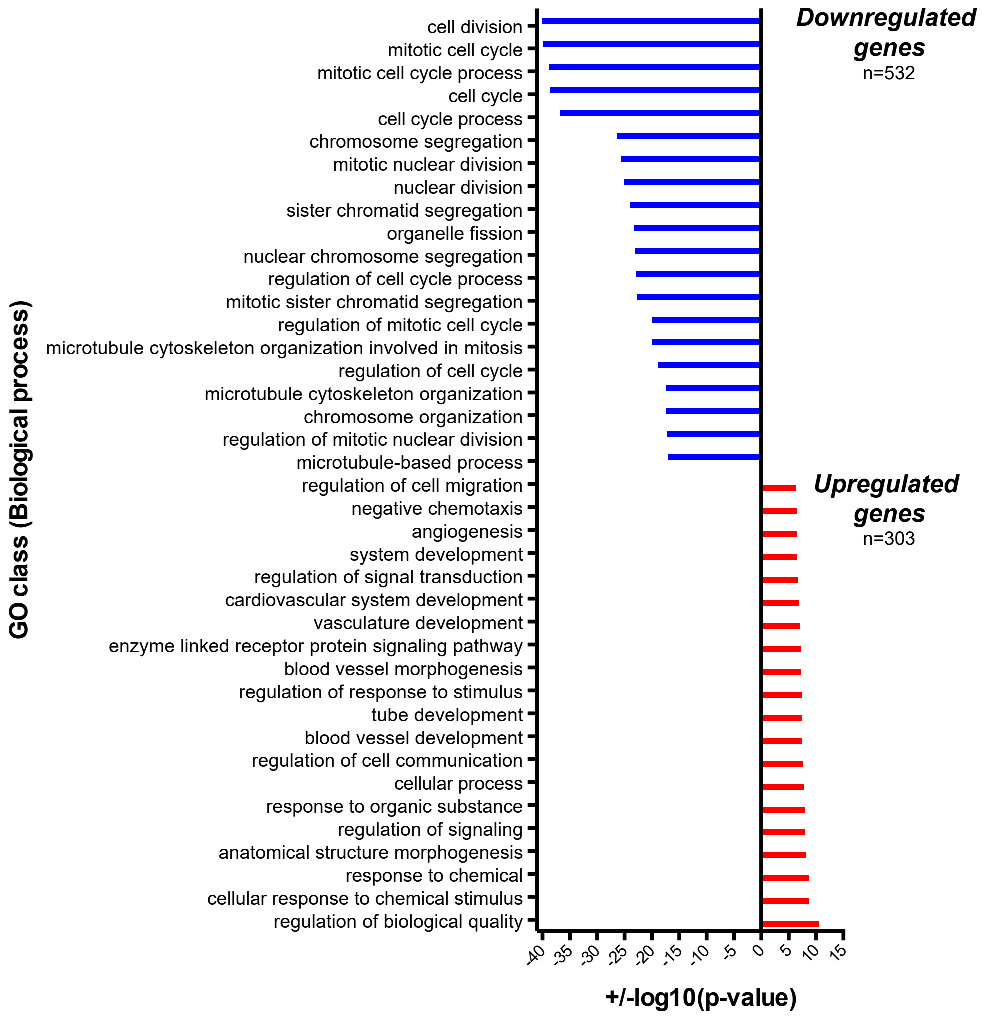

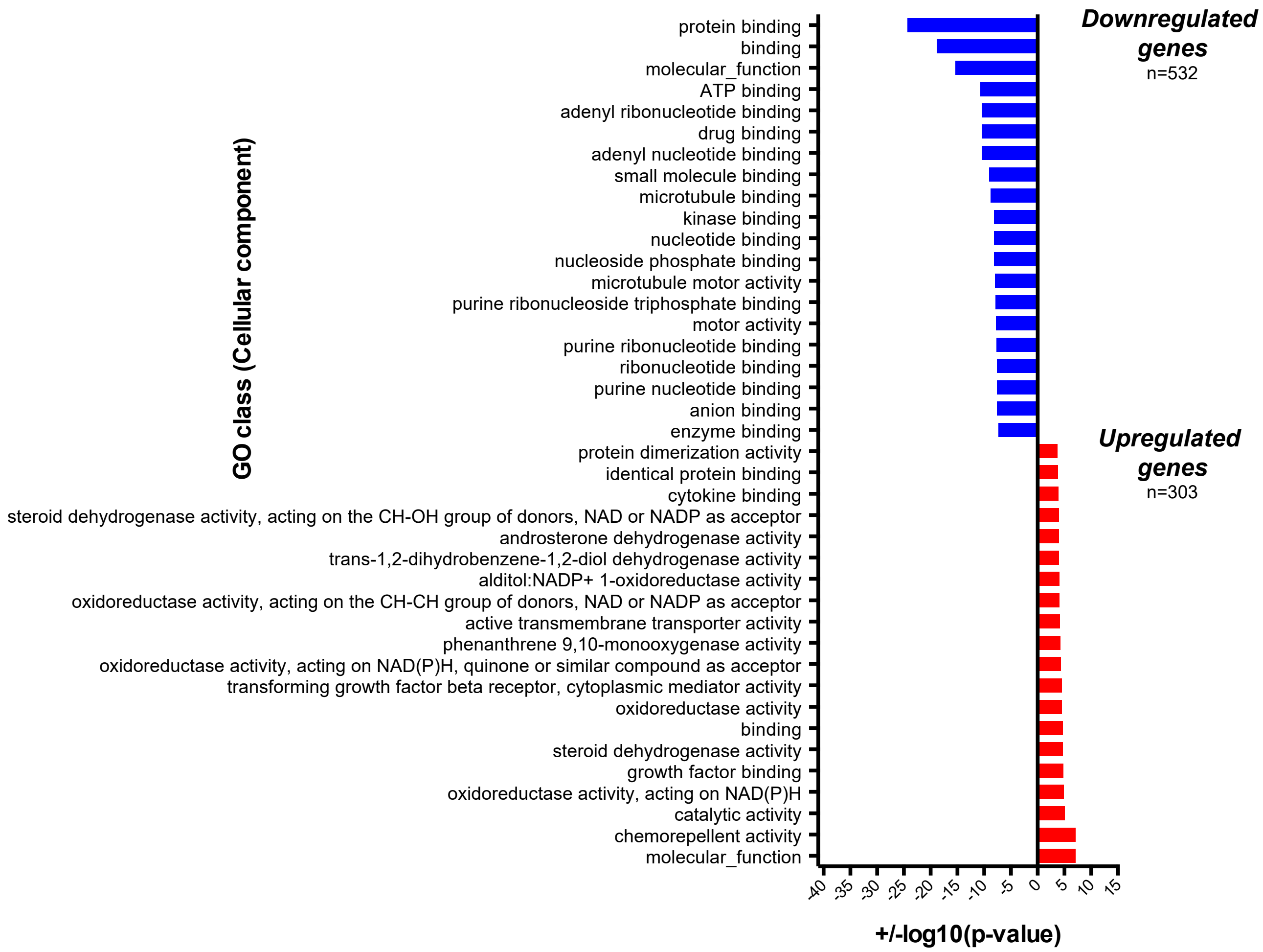

Supplementary Figure 4 - Follain_Osmani et al.

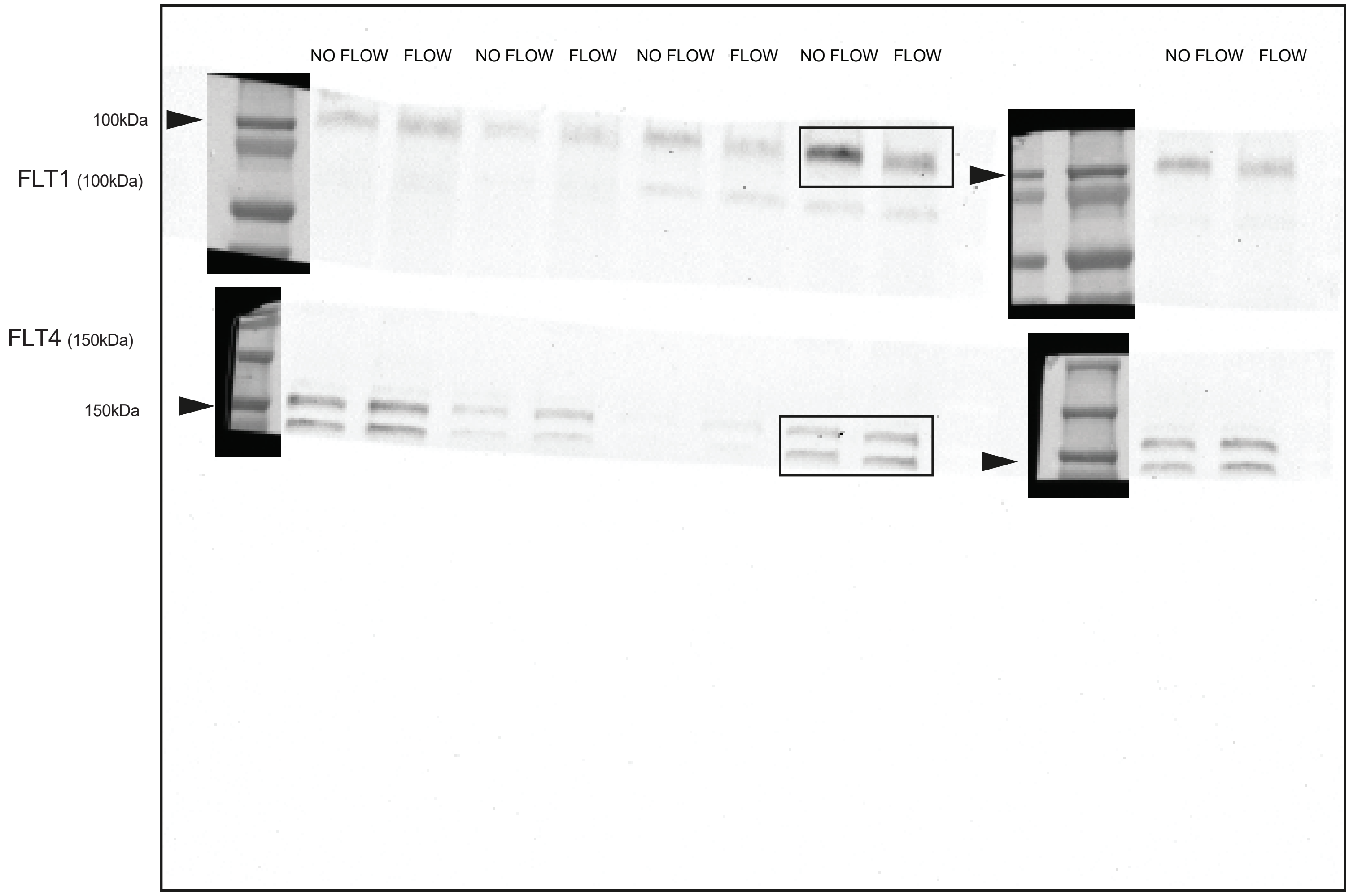

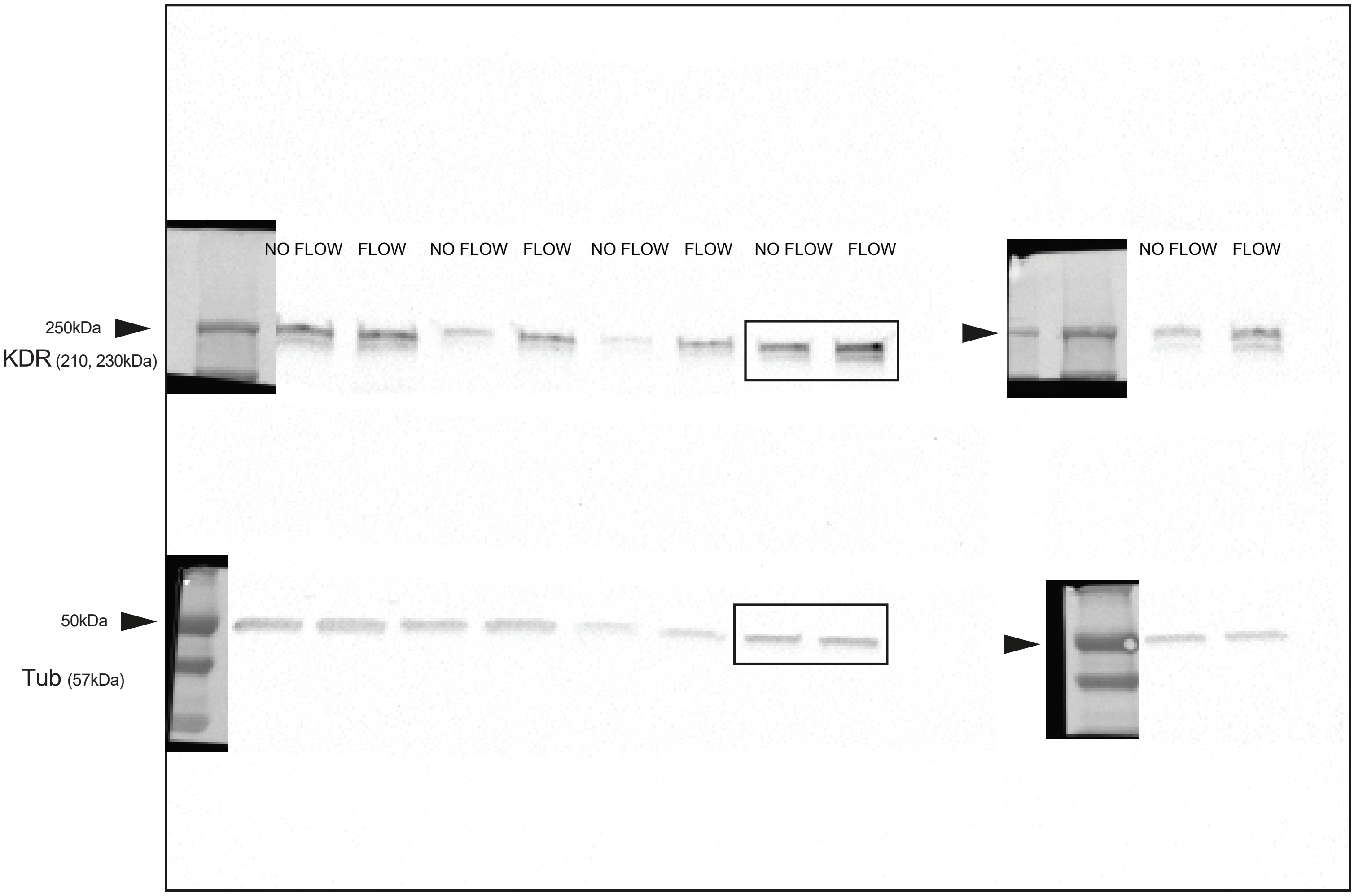

Supplementary Figure 5 - Follain_Osmani et al.
